# The European Nucleotide Archive in 2017

**DOI:** 10.1093/nar/gkx1125

**Published:** 2017-11-13

**Authors:** Nicole Silvester, Blaise Alako, Clara Amid, Ana Cerdeño-Tarrága, Laura Clarke, Iain Cleland, Peter W Harrison, Suran Jayathilaka, Simon Kay, Thomas Keane, Rasko Leinonen, Xin Liu, Josué Martínez-Villacorta, Manuela Menchi, Kethi Reddy, Nima Pakseresht, Jeena Rajan, Marc Rossello, Dmitriy Smirnov, Ana L Toribio, Daniel Vaughan, Vadim Zalunin, Guy Cochrane

**Affiliations:** European Molecular Biology Laboratory, European Bioinformatics Institute, Wellcome Genome Campus, Hinxton, Cambridge, CB10 1SD, UK

## Abstract

For 35 years the European Nucleotide Archive (ENA; https://www.ebi.ac.uk/ena) has been responsible for making the world’s public sequencing data available to the scientific community. Advances in sequencing technology have driven exponential growth in the volume of data to be processed and stored and a substantial broadening of the user community. Here, we outline ENA services and content in 2017 and provide insight into a selection of current key areas of development in ENA driven by challenges arising from the above growth.

## INTRODUCTION

In June 1982, the European Molecular Biology Laboratory (EMBL) published the EMBL nucleotide sequence data library comprising 565 sequence records. In the intervening years, there have been significant advances in sequencing technology. Improvements in automation for DNA sequencing (1,2), leading toward the first commercial DNA sequencers (3), made sequencing more accessible and paved the way for achievements such as the draft human genome published in 2001 (4,5). Introduction of the first next-generation sequencing platform in 2005 (6) continued to drive down the cost of sequencing, in terms of both time and money, and increase the feasibility of whole genome projects (7). While these, and on-going, advances in the field have proven to be invaluable to the scientific community, they have also brought challenges to the public sequence databases. Data growth requires continuous technical development and our services face a user community of growing size and diversity, requiring new approaches to user support and greater automation in data validation and processing. Today, 35 years after the first release, the database now stands at over 800 million public assembled and annotated sequence records, with such sequences only one of the many data types managed by the European Nucleotide Archive (ENA), maintained at the EMBL European Bioinformatics Institute (EMBL-EBI).

Managing the world’s sequencing data is achieved in collaboration between EMBL-EBI (ENA) and partner operations at the National Center for Biotechnology Information (NCBI GenBank (8) and Sequence Read Archive) and the DNA Databank of Japan (DDBJ) (9), together forming the International Nucleotide Sequence Database Collaboration (INSDC; http://www.insdc.org) (10). Data are exchanged routinely between partners to ensure globally comprehensive data coverage and to maximize availability of sequence data, with INSDC partners sharing responsibility for services and support for data submission and access.

In this article, we first outline ENA content and services in 2017. We then turn to a selection of areas of active development at ENA in which we are currently responding to the challenges of growth in order to ensure continued provision of our sequence data resource into the future.

## SUMMARY OF ENA CONTENT AND SERVICES

In 2017, ENA has continued to provide comprehensive services freely to the community for the submission, archiving and presentation of sequence data across a spectrum of data types. Submission services, operating through the Webin interactive and programmatic interfaces (https://www.ebi.ac.uk/ena/submit/sra/#home), continue to support several thousand active data submitters through provision of an expert helpdesk (https://www.ebi.ac.uk/ena/support/contact). Secure and permanent data archiving is provided based on the EMBL-EBI’s technical infrastructure. Data presentation services include the ENA Browser (https://www.ebi.ac.uk/ena) for web-based and RESTful programmatic data retrieval; various search interfaces including Advanced search (https://www.ebi.ac.uk/ena/data/warehouse/search), sequence similarity search (https://www.ebi.ac.uk/ena/data/sequence/search) and the Discovery API (https://www.ebi.ac.uk/ena/portal/api/); and a range of services providing high-volume data access such as the ENA File Downloader (https://github.com/enasequence/ena-ftp-downloader) and the assembled/annotated sequence FTP site (ftp://ftp.ebi.ac.uk/pub/databases/ena). Extensive documentation is available from our website (https://www.ebi.ac.uk/ena/about) and we are increasingly recommending our growing range of user tutorials (https://ena-docs.readthedocs.io/en/latest/index.html). ENA content spans raw read data from a variety of platforms and derived from over 3 million libraries, served in FASTQ and, where provided, CRAM formats; around 500 000 read alignments and re-alignments; levels of assembly from contigs through to completed closed chromosome (over 140 000 assemblies); approaching 900 million assembled/annotated sequences; around 250 taxonomic/functional identifications on metagenomics data; and a layer of contextual data and metadata relating to sampling processes, experimental design and bioinformatics processing (for example, 2.7 million sample records).

## AUTOMATING SEQUENCE SUBMISSIONS

**Figure 1.**
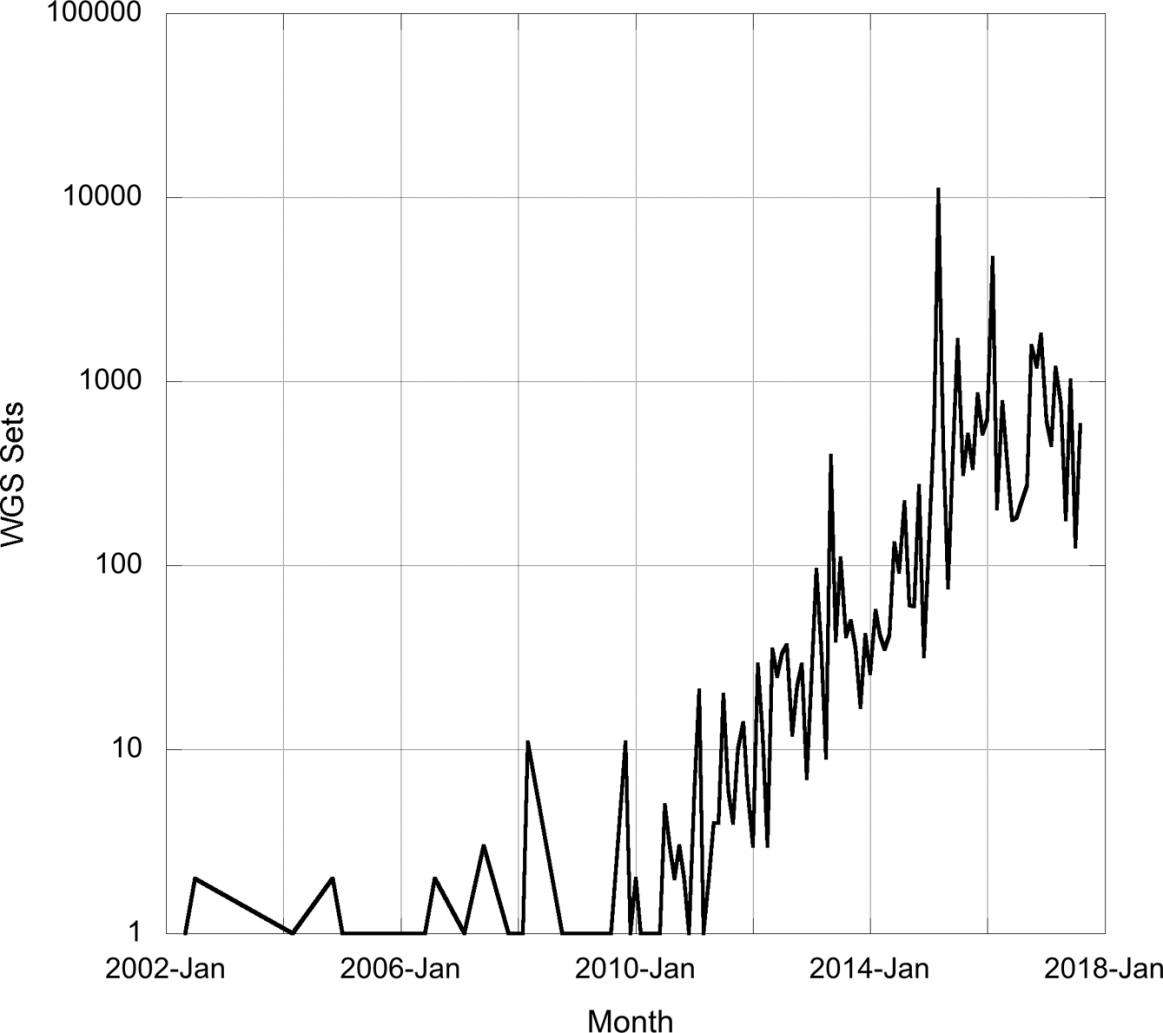
Number of WGS sets accessioned each month at ENA.

Over the years there have been several methods (and services) for submitting assembled and annotated sequences to ENA, all requiring manual processing, including data integrity validation, of the records by EMBL-EBI staff before assignment of accession numbers. With growing rates of submission of this data type, manual processing was set to become unsustainable. Submission processing for whole genome shotgun contigs (WGS) and genome assembly sequences was automated in early 2014 (11), drastically increasing our ability to handle the growing volumes of submissions (Figure 1). Following introduction of this system, in the majority of cases it is possible to accession sequences without human intervention and yet retain validation of data integrity.

With similar submission-related challenges arising for non-WGS sequences, we continued, as part of our ‘Sustainable Biocuration’ program (12), to build on the success of the WGS and genome assembly submission system to include all assembled and annotated sequences. In June 2017, we launched a significant new component to our Webin submission framework (https://www.ebi.ac.uk/ena/submit/sra/#home) to this end. With this change, all major classes of assembled/annotated sequence data are routed through a workflow that is autonomous with respect to EMBL-EBI staff (Transcriptome Shotgun Assembly (TSA) and Third Party datasets (TPA) are the only remaining data classes excluded from these new processes). This brings the vast majority of users through a single submission technology base and provides a more rapid turnaround from submission to accessioning and presentation in ENA.

The new submission service requires submitters to choose from a list of sequence annotation ‘checklists’. These are the same checklists as were previously available in the outgoing system (https://www.ebi.ac.uk/ena/submit/annotation-checklists), so regular submitters will experience a familiar submission process. Both sequences and their associated functional annotations are submitted according to the checklists in tab-separated (TSV) format and are transformed into flat files for archiving and presentation. Both interactive and programmatic interfaces are provided, with the latter also supporting submission of pre-prepared flat files. The interactive submissions interface also provides the option to build a submission using a web form. The submission process is fully automated with respect to EMBL-EBI staff, including data integrity checks, functional annotation validation and data presentation, bringing the advantages of faster turnaround times from submission to accessioning and greater consistency between annotations.

A major difference in the new service is the requirement that all sequences are associated with a study record. The study record serves to group sequences for control of the data release process. It is therefore recommended that all sequences for a single publication, or to be released publicly at the same time, should be registered under a single study, regardless of sequence type; this may be one study per submission or one study for several submissions. Any studies registered by the submitter are saved into their Webin account and made available for re-use within the system.

## OPTIMIZING HIGH-VOLUME ASSEMBLED/ANNOTATED SEQUENCE RELEASE

**Figure 2.**
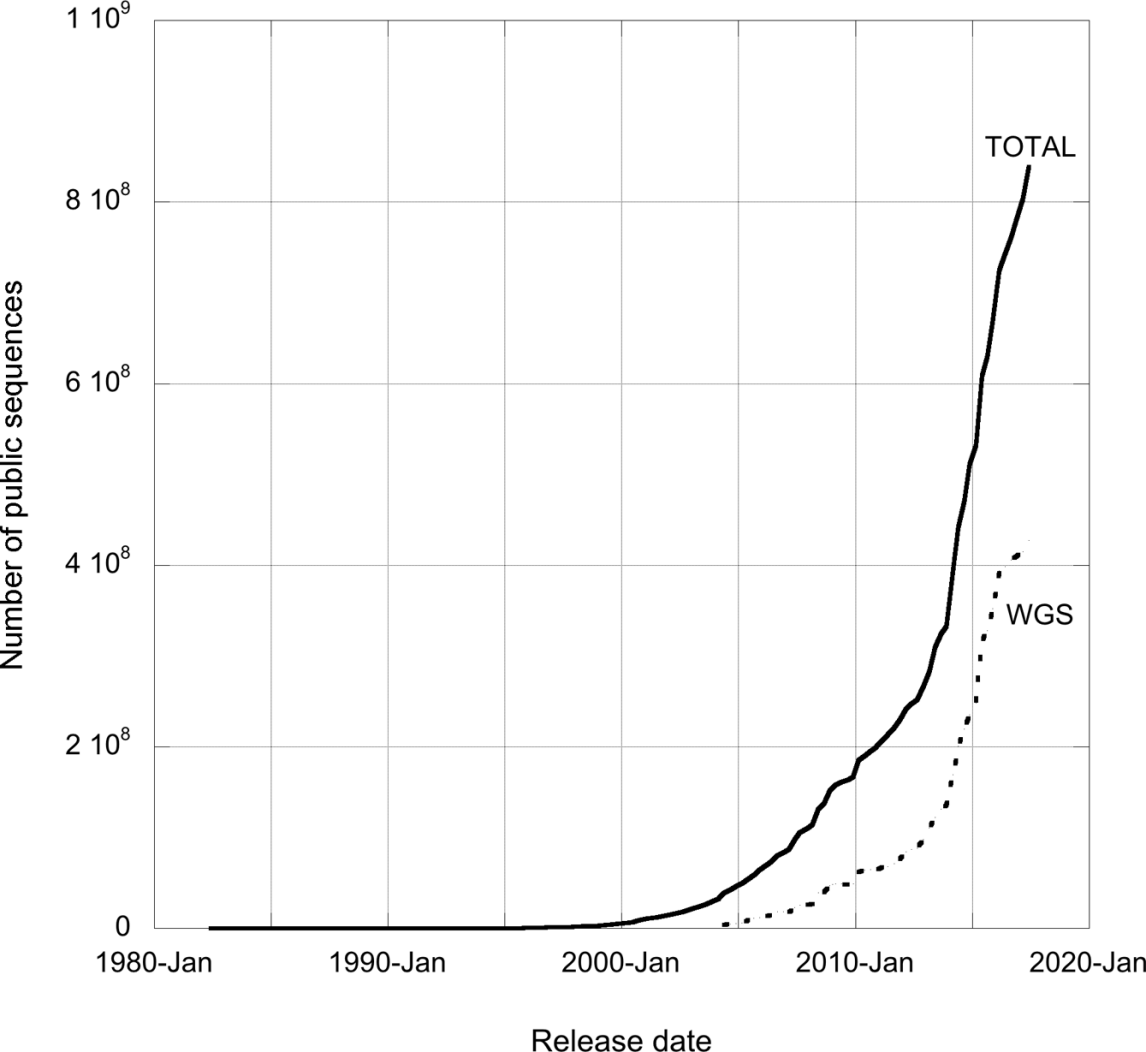
Total number of public sequences contained in each release of ENA’s assembled and annotated sequence database from June 1982 to June 2017. From June 2004, when WGS sequences were first included in the release, the WGS proportion is also illustrated.

The number of records in the assembled and annotated sequence database release has been increasing exponentially since the beginning (Figure 2). This was exacerbated with the addition of WGS sequences into the release in 2004. The 132nd release produced in June 2017, 35 years after the first release, contained 838 955 095 entries, over half of which were WGS sequences (427 307 198 entries); it took ∼4 weeks to generate and required over 10 000 h on the EMBL-EBI computational cluster. The resource cost, in terms of people, computational time and load on databases and file systems, is not limited to the generation of the sequence files, but includes also the processing of these files to make the records available for search and retrieval within the ENA Browser. We have identified two areas that can be addressed to handle this ever-increasing data volume: reducing the size of the release and optimizing data distribution methods.

### Reducing the size of the release: separating out sequence sets

Originally, sequences submitted to the INSDC archives were treated as single entities. The idea of grouping sequences into sets was introduced first with WGS and later with TSA. Sets are given a single accession prefix and updates to the sequences tend to be presented as an update to the entire set, with an increment of the build number (captured in the digits immediately after the prefix) for the set. When users retrieve WGS and TSA sequences, they tend to be interested in the set as a whole, rather than in individual sequences within the set. Despite these ideas of treating a set of sequences as a single entity, for many years data distribution procedures have handled sets in the same way as non-set sequences; WGS and TSA sequences were grouped per set in the release and update FTP locations, but beyond this their set-based properties did not feature in data distribution. The biggest impact of this was the need to regenerate (and reprocess) a set at every release, regardless of whether there had been any changes made to the set. Given the high proportion of sequences that are contained within these sets, this led to a high level of redundant computation.

In early 2017, we introduced new FTP locations for WGS and TSA sequences (ftp://ftp.ebi.ac.uk/pub/databases/ena/wgs and ftp://ftp.ebi.ac.uk/pub/databases/ena/tsa, respectively). These allow users to retrieve WGS and TSA sequence sets from a single location without requiring knowledge as to whether the set has been updated or published since the last release. At the same time, we made all suppressed sets (and builds) available for easy download; the above FTP locations each have two sub-directories: public and suppressed. We provide an EMBL flat file format master record for the set presenting annotation common to all sequences in the set, a compressed EMBL flat file format file containing all sequences in the set and a compressed file containing the sequences in FASTA format. These files are updated as part of our daily distribution cycle and a traditional release-style update is only performed when there has been an EMBL flat file format change (currently no more than once a year). All WGS and TSA set sequences will be removed from the release upon the first release of 2018 and users will be directed to these new FTP locations. Those TSA sequences that were accessioned prior to the introduction of sequence sets for TSA will continue to be distributed as before with each release.

### Optimizing data distribution

Long running projects come with the very real danger of spawning unwieldy and difficult to maintain code bases. As the years go by, the code passes through the hands of many developers who each need to add functionality as requirements change and new data types are added. In addition to the human resource costs involved in running workflows built in this way, the processes themselves perform sub-optimally. With the distribution of assembled and annotated sequences spanning four decades, assembled/annotated sequence distribution at ENA falls into this category, bringing the need for refactoring every few years.

Over the last 2 years our refactoring work has seen us revisiting the different components involved in sequence publishing workflows, from selecting which sequences need to be distributed to writing EMBL flat files into distribution products. In some cases this has involved a simple reworking and tuning of SQL queries, while in other cases it has required the complete rewrite of a part of a workflow, often including a change in programming language. By the end of 2017, we will have constructed a new daily update workflow with these new building blocks and in early 2018 we will start exporting this work onto quarterly sequence release processes.

## SCALING THE SEQUENCE VERSION ARCHIVE (SVA)

**Figure 3.**
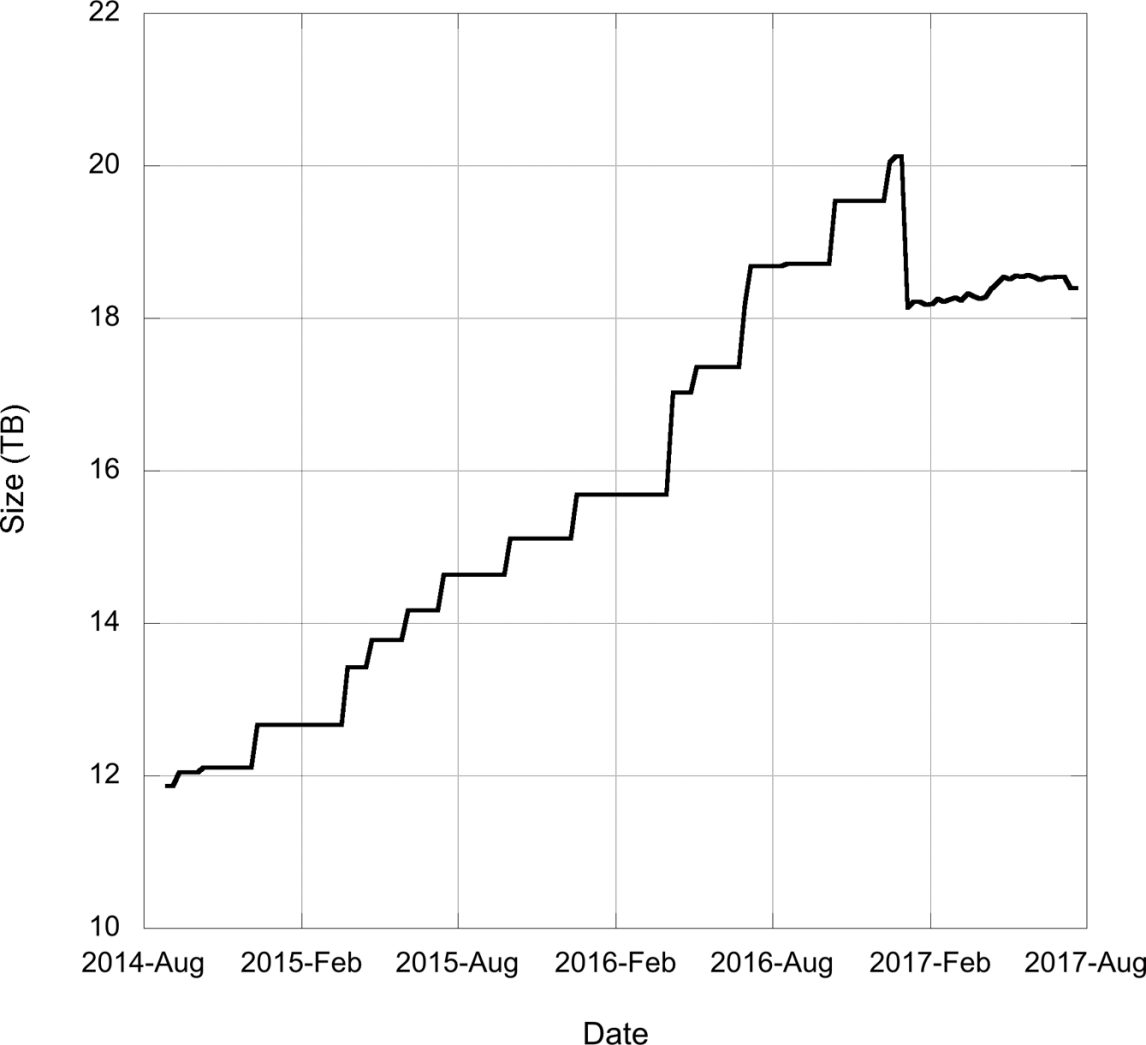
Recent growth in the Sequence Version Archive database. This is given as the compressed data size.

ENA offers services for users to access suppressed and replaced sequences and annotation through the Sequence Version Archive (SVA), introduced in 2003 (13). The service is built using an Oracle database that holds a copy of every incremental change to a sequence record. With every change, even a minor edit such as an update to a cross-reference, a new copy is saved into the database. Together with exponential sequence growth, the practice of saving every change resulted in unsustainable growth in database size, with impacts upon our ability to backup the database and run queries (Figure 3). We have therefore sought new approaches that retain the value of the SVA service, while constraining growth of the underlying database.

Short-term measures have been appropriate to limit growth while we put in place our long-term solution. Expanded CON (constructed) record loading has always been limited to daily updates and one release per year due to size of this data collection. In late 2016, we removed support for expanded CON records (which can be recalculated at any point from their underlying contig sequences) and stopped loading WGS and TSA sets, resulting in a database size reduction of ∼2TB and slower subsequent growth (Figure 3). Our longer-term solution required consideration of two areas: the choice of which versions of records should be saved (i.e. which changes are significant in meaning and justify storage) and a more suitable database technology.

### Defining the set of records that need to be stored

Storing all incremental changes to a sequence record can result in unnecessarily inflated data volume. For example, human chromosome 1 (CM000663) currently only has two sequence versions (CM000663.2 is public; https://www.ebi.ac.uk/ena/data/view/CM000663) however there are 17 copies of this record in the SVA capturing often very minor changes, typically to the syntax (rather than the semantics) of annotation. We investigated the usage of SVA and found that, in the majority of cases, historical annotation changes were not of interest to users; rather, sequence versions were. Moving toward a model where annotation and cross-reference changes within a sequence version results in a replacement of the record stored, rather than the addition of a new one, drastically reduces the volume of data to be stored.

Retrieval of sequences from SVA has always been limited to one sequence at a time, restricting the usability for WGS and TSA sets. With the introduction of suppressed WGS and TSA available for download via FTP (see above), there is no further need for WGS and TSA sequence sets to be saved within SVA. Deleting the WGS and TSA sequences, together with the historical annotation change records, reduces the SVA database size from over 18TB to just over 1TB. This new volume takes the database well into the acceptable size range, both in terms of database administration and performance of the services in which we wish to provide. This also allows us to revisit expanded CON sequences and we plan to add support for these records back into the database as part of the redesign process.

### MongoDB: a more appropriate file store database

The original design of the SVA service uses a database as a file store, a design feature that will be retained. However, currently an Oracle database is used. While this may have been a suitable option at the time of creation, with advances in database technologies in the intervening years, better solutions have become available. We have chosen to replace the SVA Oracle database with MongoDB technology. MongoDB is a non-relational database that was first released in 2009. Its key benefits include flexible metadata structures, the distributed nature of the database to provide high availability and scalability and a purpose-built module to support the use of the database as a file store. These features were critical in our decision to build the new SVA around MongoDB. The ability to combine searchable metadata with stored sequence record ‘files’ within the same database means that we can take several legacy workflows that support both the existing SVA and ENA Browser services and merge them into one. This will result in a reduction in both storage requirements and computational resources as we transition from a model where multiple copies of data are each processed for multiple purposes to one in which a single copy of the data is processed once.

### Timeline for SVA changes

The planned changes to the SVA are being carried out as part of larger initiative that impacts also the ENA Browser, with the plan that there will no longer be a separate service to provide access to suppressed and replaced sequences. We are still in the early stages of the SVA redesign and will announce when new services become available via our news page (https://www.ebi.ac.uk/ena/news), announcements mailing list (http://listserver.ebi.ac.uk/mailman/listinfo/ena-announce) and both the EBI and ENA twitter accounts (@emblebi and @enasequence, respectively). We will maintain a period of overlap where both old and new services can be used and anticipate that the existing SVA will be retired mid-2018.

## SELECTED KEY DEVELOPMENTS IN 2017

It is important to ensure that all scientists wishing to submit their sequence data to ENA can do so with relative ease. We therefore drive at simplification of submission services as an on-going goal, while balancing the need for integrity in incoming data and adequate description to allow integration into ENA to provide ultimate discoverability, interoperability and reusability. It is therefore imperative to have clear instructional documentation. To this end, we have been developing a new suite of tutorials, which currently include one each for interactive and programmatic submissions (http://ena-docs.readthedocs.io/en/latest/).

With the expanding scope and portfolio of ENA it is necessary to contract some areas in order to restrict the maintenance burden as well as to free resources for expanding high use/cost services. As part of this work, we have introduced the ENA Data Discovery API (https://www.ebi.ac.uk/ena/portal/api/). Based on the same concepts, but simplifying and extending the functionality (Table 1), this new system will eventually replace the existing Advanced Search for programmatic users. The API will support many new requirements including, where users request it, the sharing of pre-publication data within collaborating groups. For this reason, the discovery API requires authenticated access. Anonymous access is supported for searches of public data only. With changes made to the underlying database, we have also been able to add support for combined data-sample searches, a much-requested functionality. Full documentation for the API is available at https://www.ebi.ac.uk/ena/portal/api/doc.

**Table 1.** Main simplifications and changes in use of the new data discovery API compared to ENA’s advanced search

Parameter	Change
query	This is now optional. If this parameter is not supplied, the full result set for the selected data type will be returned.
domain	This parameter is no longer needed/supported.
offset	This has been changed to the true offset and represents the number of records to skip rather than the number of the record from which to start the result page.
limit	The default remains at 100 000 records, but this can now be set to 0 to fetch all records for the search.
length	This parameter is no longer needed/supported.
format	Only metadata reports are currently supported therefore the meaning of this field has changed. This parameter directs whether the search report should be downloaded in TSV (default) or JSON format.
dataPortal	The API introduces the concept of data portals to support additional services to ENA. The current selection is: ena, pathogen, faang and metagenome.
